# Describing socio-economic variation in life expectancy according to an individual's education, occupation and wage in England and Wales: An analysis of the ONS Longitudinal Study

**DOI:** 10.1016/j.ssmph.2021.100815

**Published:** 2021-05-08

**Authors:** Fiona C. Ingleby, Laura M. Woods, Iain M. Atherton, Matthew Baker, Lucy Elliss-Brookes, Aurélien Belot

**Affiliations:** a Department of Non-Communicable Disease Epidemiology, Faculty of Epidemiology and Population Health, London School of Hygiene and Tropical Medicine, London, UK; b School of Health & Social Care, Edinburgh Napier University, Edinburgh, UK; c National Cancer Research Institute Consumer Forum, London, UK; d National Cancer Registration and Analysis Service, Public Health England, London, UK

**Keywords:** Life expectancy, Mortality, Socio-economic status, Income, Educational status, Occupational groups, Census data

## Abstract

People who live in more deprived areas have poorer health outcomes, and this inequality is a major driver of health and social policy. Many interventions targeting these disparities implicitly assume that poorer health is predominantly associated with area-level factors, and that these inequalities are the same for men and women. However, health differentials due to individual socio-economic status (SES) of men and women are less well documented. We used census data linked to the ONS Longitudinal Study to derive individual-level SES in terms of occupation, education and estimated wage, and examined differences in adult mortality and life expectancy. We modelled age-, sex- and SES-specific mortality using Poisson regression, and summarised mortality differences using life expectancy at age 20. We compared the results to those calculated using area-level deprivation metrics. Wide inequalities in life expectancy between SES groups were observed, although differences across SES groups were smaller for women than for men. The widest inequalities were found across men's education (7.2-year (95% CI: 3.0–10.1) difference in life expectancy between groups) and wage (7.0-year (95% CI: 3.5–9.8) difference), and women's education (5.4-year (95% CI: 2.2–8.1) difference). Men with no qualifications had the lowest life expectancy of all groups. In terms of the number of years' difference in life expectancy, the inequalities measured here with individual-level data were of a similar magnitude to inequalities identified previously using area-level deprivation metrics. These data show that health inequalities are as strongly related to individual SES as to area-level deprivation, highlighting the complementary usefulness of these different metrics. Indeed, poor outcomes are likely to be a product of both community and individual influences. Current policy which bases health spending decisions on evidence of inequalities between geographical areas may overlook individual-level SES inequalities for those living in affluent areas, as well as missing important sex differences.

## Highlights

• Health policy to reduce inequalities targets individuals, although often based on area-level data.
• We show wide inequalities in health outcomes using individual-level socio-economic data.
• Individual-level inequalities are of a similar magnitude to those observed with area-level data.
• We find important sex differences in inequalities which are undetectable in area-level analyses.
• Both individual and area-level factors should be considered in policy aimed at reducing inequality.

## Introduction

In many high-income countries, people who live in socio-economically deprived localities have higher all-cause mortality than those living in more affluent localities (Bennett et al., 2018; Butler et al., 2010; Rey et al., 2009; Singh & Siahpush, 2002; Woods et al., 2005). The comprehensive documentation of this trend in the UK has rightly motivated an increased policy focus on reducing inequalities (Marmot et al., 2021). The importance of these inequalities has more recently been elevated within the NHS (National Health Service) long-term plan for 2020–2030 (NHS, 2019); the latest re-imagining of NHS priorities for England. Within this refreshed policy, funding is directly linked to measured socio-economic inequalities, now being based upon “a *more accurate assessment of health inequalities and unmet need*” whilst all major national programmes and local areas now have a specific responsibility *“to set out specific measurable goals and mechanisms by which they will contribute to narrowing health inequalities over the next five and ten years”.*

These measured inequalities, along with the majority of research relating to socio-economic differentials of health in the United Kingdom, are largely based upon measures of ‘ecological’ deprivation differentials that use administrative data aggregated to small areas, mainly due to pragmatic reasons such as data availability. The Index of Multiple Deprivation (IMD) is the most commonly used index to estimate area-level deprivation in the UK. This index is calculated as a weighted score combining sub-indices that include important socio-economic variables such as income, education, and employment (Office for National Statistics, 2020). There is evidence to suggest that inequalities in mortality and life expectancy are also found across individual-level socio-economic measures, for example, increased life expectancy was shown to be associated with increased personal income in the US (Chetty et al., 2016); socio-economic inequalities in the relative risk of death were found across several European countries (Mackenbach et al., 2008); and inequalities in life expectancy for those aged over 50 have been described in England (Parker et al., 2020). Individual-level analysis offers an interesting perspective by quantifying mortality differences in terms of individual socio-economic status (SES), as opposed to the ecological differences between different geographical areas, which quantifies the impact of ecological (i.e. contextual) deprivation on the health outcome of interest. Policies aimed at reducing inequalities will benefit from a deeper understanding of health outcomes in terms of individual socio-economic status, which has not been quantified in detail for working-age adults in a recent UK population setting. Clearly there are practical issues that will influence whether health policies target individualised or area-level inequality issues; nonetheless, further research will be especially important given the recent context of an increasing focus on individualised care within the NHS, as well as recent support for the potential to tackle socio-economic inequalities via increased attention to individual circumstances (Moscrop et al., 2020).

We have recently shown that the concordance between aggregated deprivation metrics at a small-area level and the observed individual SES can in fact be relatively low, such that describing small-area populations with only aggregated statistics will tend to overlook significant numbers of deprived individuals who reside outside deprived areas (Ingleby et al., 2020). We also demonstrated that the relationship between an individual's socio-economic status and area-based deprivation differs by sex, which is ignored by the use of area-based metrics which combine data from men and women. In short, these findings show that there are significant numbers of people whose individual socio-economic status does not match that of the area they live in, which raises the possibility that health policy aimed at reducing inequalities via area-level interventions may have limited effectiveness, as has already been demonstrated for cancer (Rachet et al., 2010), whilst important disadvantages experienced by men and women of particular SES living in less deprived areas may go under the radar.

Where inequalities in mortality between individual-level socio-economic groups exist, they are likely to have distinct underlying causes to inequalities observed between more and less deprived areas. For example, while higher mortality in more deprived areas could be addressed via area-based interventions such as changes to the distribution of healthcare resources; higher mortality in, for instance, individuals with lower educational attainment, might be tackled via changes in the methods and routes used for public health communication. An improved understanding of mortality differences across individual socio-economic circumstances and how they compare to inequalities observed using aggregated data is therefore necessary to inform which specific changes in policy (area-based or individual-level) could be more effective in targeting the underlying causes.

Here, we take an initial step in this direction by first describing the patterns and extent of socio-economic inequalities in mortality and life expectancy between individuals grouped according to their education, occupation, and (estimated) wage. We then compare these estimates to existing measures which use aggregated area-level deprivation metrics (Bennett et al., 2018; Woods et al., 2005). We discuss the meaning of these results in the context of implications for the funding and focus of healthcare policy aimed at reducing inequalities.

## Methods

**Fig. 1:**
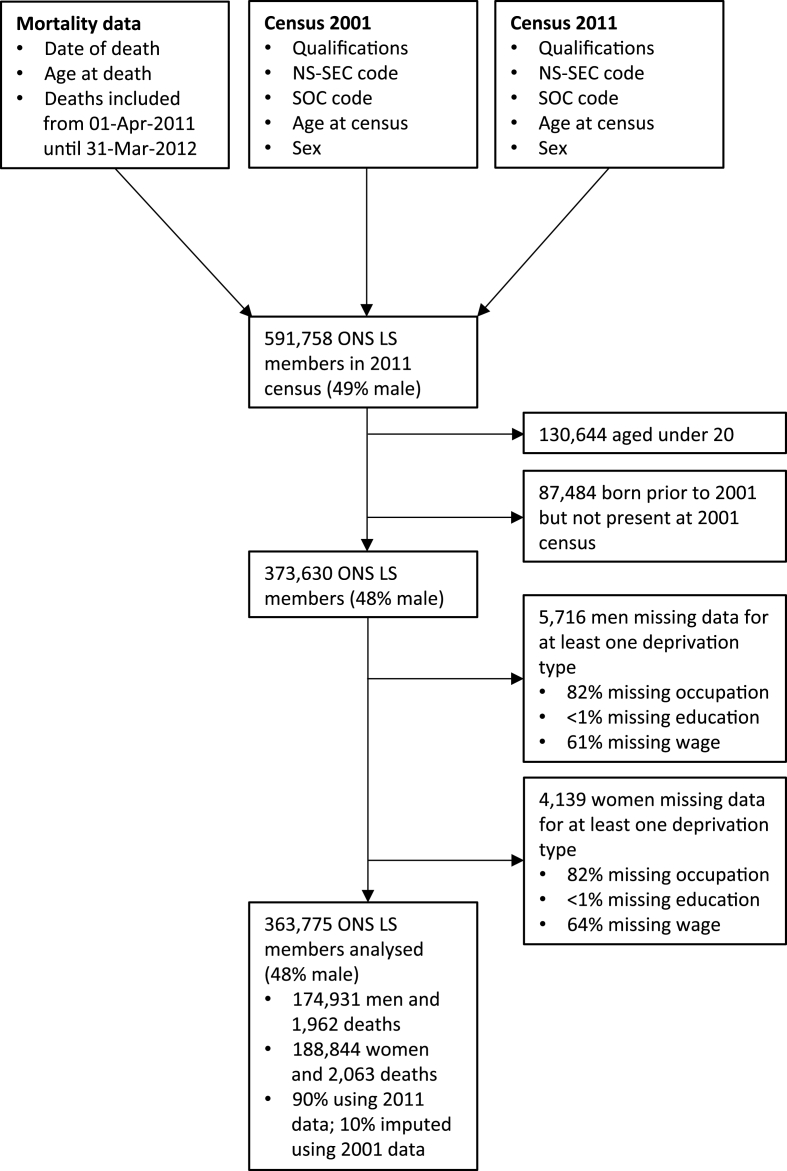
Consort diagram describing the dataset linkage and variables used in the analysis, as well as the flow of LS members through the data processing steps: overall numbers, analysis cohort filtering, and missing data exclusions. Data source: ONS LS.

We examined the Office of National Statistics Longitudinal Study (LS) (Shelton et al., 2019; Hattersley & Creeser, 1995), a long-term cohort study comprised of people living in England and Wales under selection criteria of one of four annual birthdates (representing a random sample of approximately 1% of the population clustered by date of birth). All census variables from the 1971 census through to the most recent 2011 census are directly linked to cohort members via unique identifiers, and additional variables are also derived via individual linkage, including administrative data such as births and deaths. We included LS members enumerated at the 2011 census (the most recent census to have taken place) and linked to mortality data to include deaths in the 12-month period subsequent to the census (i.e. 01-Apr-2011 to 31-Mar-2012). Age, sex, and data relating to occupation and educational qualifications for 2001 and 2011 censuses were extracted (Fig. 1) and used to categorise LS members according to three dimensions of individual-level socio-economic circumstances: occupation, education, and wage. These measures are often used to summarise overall SES (Conway et al., 2019; Winkleby et al., 1992) but here we are analysing these three measures independently rather than using a combined score.

### Deriving individual socio-economic variables

Data from the 2011 census were used as the main source of information for individual socio-economic status. Missing 2011 census data were completed where possible by proxy, using data as reported for household head in the majority of cases; or if unavailable, another adult resident. If no data was available for these people in the 2011 census, data were completed from the 2001 census, which was possible where the LS member was enumerated at both time points. Initially, socio-economic data was missing for 11%, 1% and 9% of individuals for occupation, education and wage, respectively; after completion of missing data by household adult proxy, missing data was 2%, <1% and 2% for occupation, education and wage.

For occupation, the three-category version of the National Statistics Socio-Economic Classification (NS-SEC) (Office for National Statistics, 2010) was applied, grouping occupations as *technical, routine and manual occupations*; *intermediate occupations*; or *higher managerial, administrative and professional occupations*. As recommended for the three-category version of the NS-SEC (Office for National Statistics, 2010), those without an occupation classification due to long-term unemployment or studentship were treated as missing and therefore completed with another household adult as a proxy where possible, as described above.

Individual education was defined on the basis of qualifications recorded at census. The standard derived variable for highest qualification (Centre for Longitudinal Study Information and User Support, 2020) was used for each individual, which includes categories for *no qualifications*; *1–4 GCSEs or equivalent*; *5* + *GCSEs or equivalent*; *apprenticeships and vocational qualifications*; *A-levels or equivalent*; and *degree-level education*. These represent standard qualifications earned in English and Welsh education, with GCSEs gained first and A-levels later, or the equivalent level of qualification for individuals educated within a different educational system outside England and Wales.

Wage is not directly reported in the UK census. We estimated individual wage using the method derived by Clemens and Dibben (Clemens & Dibben, 2014). This approach uses an individual's sex, age and Standard Occupational Classification (SOC) code, combined with linear model coefficients, to calculate estimated wage. The method has been externally validated and the estimates found to behave very similarly to self-reported ‘real’ wage data (Clemens & Dibben, 2014). It is also worth noting that SOC codes represent far more specific and granular occupational coding compared to the NS-SEC system used to code the 3-level occupation variable, which categorises types of occupation much more broadly. As such, our wage and occupation variables are estimated independently, although it results in an expected and entirely logical correlation between an individual's type of occupation and their estimated wage. Wage estimates for those aged over 60, who are most likely to be retired, were adjusted using observed annualised percentage decreases in wage for those aged over 60 reported by the English Longitudinal Study of Ageing (ELSA) (Banks et al., 2014). The wage estimates were used to split individuals into equal quintiles within each sex, from the highest wage quintile (Q1) to the lowest wage quintile (Q5).

### Statistical analysis

Within each combination of sex and socio-economic group, individual-level data were aggregated by socio-economic category and 1-year age group to give a total number of people and a total number of deaths during the 1-year period April 2011–March 2012 for each unique combination. We derived the Person-Years (PY) as the total number enumerated at census minus half of the deaths that occurred during the following 12-month period, following standard life tables derivation methods to approximate the number of person-years for the 1-year period under analysis (Office for National Statistics, 2019a, Office for National Statistics, 2019b; Preston et al., 2000). An alternative method using exact person-years (i.e. based on calendar date of death for people who died within the 1-year period) produced very similar results.

Initial analyses used Poisson regression to estimate age-specific mortality for each sex separately (all socio-economic groups combined). Each model regressed the log of number of deaths against age at death, offset against the log of the total number of PY. For modelling the association between age and mortality rate, natural cubic splines were used with interior knots pre-specified at ages 25, 48, 60 and 75. Choice of knot locations was partly based on *a priori* knowledge of the shape of a mortality curve, combined with the results of an initial set of 200 models which included natural cubic splines of age with various combinations of randomly-generated knot locations of either 3, 4, or 5 knots (method described in (Rachet et al., 2015)). These 200 models were ranked by AIC and used to confirm choice of knot location in the final model. In addition, analyses were run to test sensitivity of the estimation method to knot location and number: while location had almost no effect, reducing the overall number of knots to only use 1 or 2 knots flattened the mortality curve and resulted in unreliable estimation. The predicted age- and sex-specific mortality rates obtained from this initial Poisson regression model were compared to those published by the ONS for the whole population of England and Wales in 2011 (Office for National Statistics, 2019a, Office for National Statistics, 2019b), as a check of the available data and method used here. This comparison highlighted unreliable estimation of mortality for individuals aged over 85 in the ONS-LS sample. Observed mortality rates with 95% confidence intervals for 5-year-interval age groups over 85 did not overlap with published mortality estimates in the whole-population life tables for England and Wales. This could be explained partly by bias introduced by a higher level of missing socio-economic data at these ages; and partly by potential immortals in the dataset (i.e. missing death records) detectable from longer life expectancy for both men and women from age 85 in the observed ONS-LS data compared to that calculated from whole-population life tables (men = 7.8 years vs. 5.8 years; women = 8.6 years vs. 6.8 years). We therefore excluded those aged over 85 from further analyses, and instead used the regression model to generate out-of-sample predictions of mortality from ages 86 through to 100. The resulting estimates using this approach were very similar to the England and Wales population estimates as published by the ONS, with 95% confidence intervals overlapping published estimates (Appendix A, Appendix A).

Next, to estimate age-, sex- and SES-specific mortality rates, the same modelling approach was carried out separately for occupation, education and wage (denoted in the equation below as the generic variable ‘SES’), by sex. Each model regressed the log of number of deaths against age x and socio-economic status (SES) (the interaction between age and SES included *a priori*) offset against the log of the total number of PY, and with pre-specified interior knots as described above for age,

$$\log\left(E\left[d_{x,\;i}\right]\right)=\;\beta_{0\;}+f\left(x\right)+\;\overset{D}{\underset{j=2}{\sum }}\beta_{j}I\left(SES_{j}=i\right)+\overset{D}{\underset{k=2}{\sum }}g_{k}\left(x\right)*I\left(SES_{k}=i\right)+\;\text{log}\left(py_{x,i}\right)$$

where $d_{x,i}$ and $py_{x,i}$ are number of deaths and PYs, respectively, for age x and socio-economic group i; I(condition) denotes an indicator variable equals to 1 if condition is true, 0 otherwise; restricted cubic spline functions are denoted f and g; and mortality is estimated for each *SES* group i=1,...,D, D being the total number of socio-economic sub-groups for the SES variable of interest (education, occupation or wage), for each sex. Mortality estimates predicted from the regression model were used to calculate life expectancy from age 20 onwards, following standard calculation methods (Preston et al., 2000) as used in the officially-published ONS estimates (Office for National Statistics, 2019a, Office for National Statistics, 2019b). Life expectancy is the average number of years of life lived from any given age, based upon the instantaneous observed mortality rates experienced in the one-year period of interest (Preston et al., 2000). Confidence intervals for the life expectancy estimates were calculated using a bootstrap method (DiCoccio & Efron, 1996): we generated 1000 bootstrap samples of the dataset, on which we fitted the models above and calculated 1000 estimates of life expectancy from age 20. The 95% confidence intervals were then reported using percentiles of the resulting distribution.

Sensitivity analysis excluding records with missing data was performed to investigate how the results would change without use of the missing data strategy described above. We compared mortality and life expectancy as a complete-case analysis and with the missing socio-economic data completed as described above, and found that the missing data strategy did not affect the results. Overall, life expectancy from age 20 estimated using the full dataset was 61.3 years for men and 63.8 years for women, compared to 62.1 years for men and 64.0 years for women when estimated as a complete-case analysis.

All statistical models were carried out in Stata v16.0, using *poisson* and *mvrs* commands (Royston & Sauerbrei, 2007) for the restricted cubic splines in the Poisson regression models.

## Results

Between 01-Apr-2011 and 31-Mar-2012, 1962 deaths amongst 174,931 men and 2063 deaths amongst 188,844 women were observed (Fig. 1). The distribution of men and women across socio-economic groups was similar, apart from with respect to the educational group ‘apprenticeships and vocational qualifications’, which had a notably higher proportion of men than women (Appendix A, Appendix A).

**Fig. 2:**
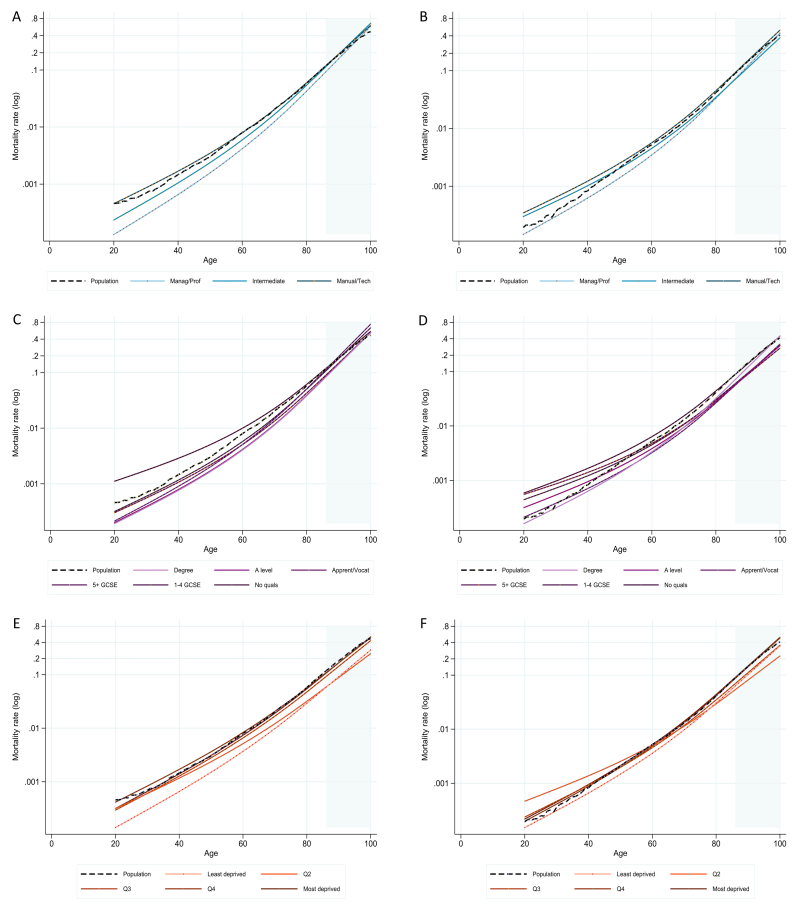
Mortality rates for 1 person-year (log scale) for ages 20-100 separated by socio-economic group and sex. In each case, the dashed black line shows the publicly-available estimates for England and Wales, 2011 (Office for National Statistics, 2019a, Office for National Statistics, 2019b). Occupation groups for men (A) and women (B); education groups for men (C) and women (D); and wage quintiles for men (E) and women (F). Shaded grey area shows ages 86–100, which were estimated by out-of-sample prediction using model coefficients (see text for details). Data source: ONS LS.

The results of models estimating age-, sex- and SES-specific mortality demonstrated considerable differences in mortality across socio-economic variables, as well as differences between men and women in terms of the type and extent of inequalities. Differences across wage quintiles and to some extent across occupational groups were relatively small for women compared to large differences seen across the same groups in men (Fig. 2), whereas the extent of differences in mortality across educational groups were more similar between the sexes.

**Fig. 3:**
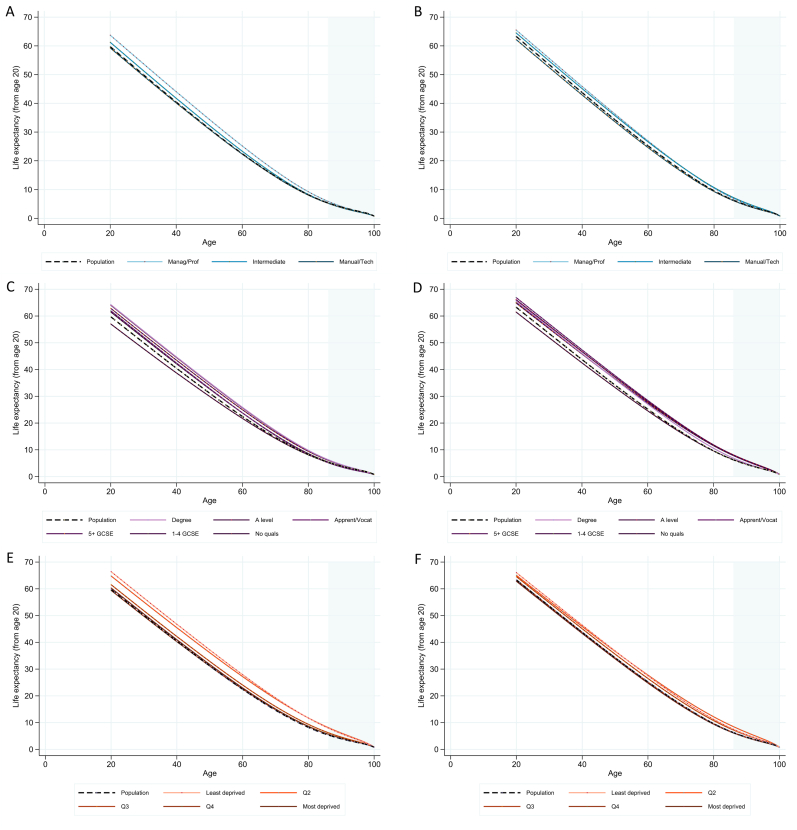
Life expectancy from age 20 calculated for ages 20-100 separated by socio-economic group and sex. In each case, the dashed black line shows the publicly-available estimates for England and Wales, 2011 (Office for National Statistics, 2019a, Office for National Statistics, 2019b). Occupation groups for men (A) and women (B); education groups for men (C) and women (D); and wage quintiles for men (E) and women (F). Shaded grey area shows ages 86–100, which were estimated by out-of-sample prediction using model coefficients (see text for details). Data source: ONS LS.

**Fig. 4:**
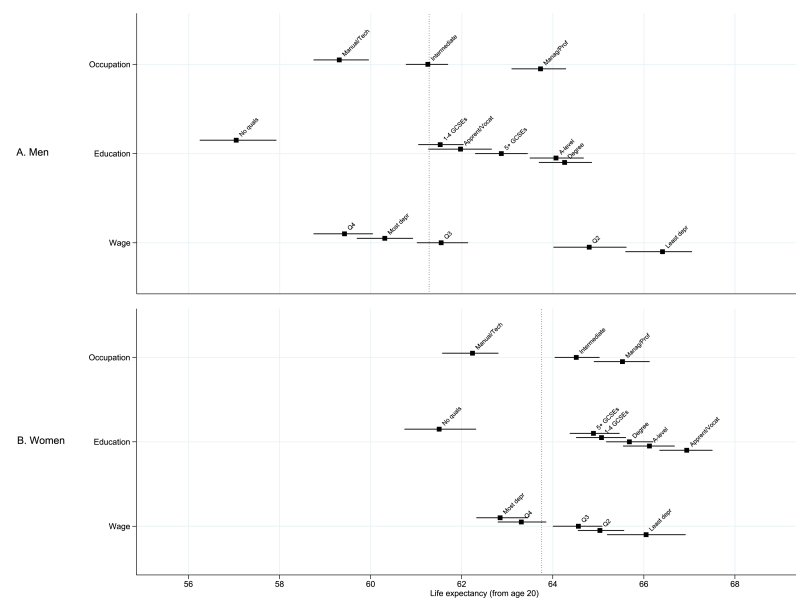
Life expectancy from age 20 (±95% CI) estimated by socio-economic group for (A) men and (B) women. Dotted line indicates life expectancy from age 20 for each sex calculated from the same data but for all socio-economic groups combined. Data source: ONS LS.

Comparison of life expectancy (calculated from age 20) between socio-economic groups and between sexes (Fig. 3) showed a fairly consistent pattern across wage and occupation groups for both men and women, with men and women in the highest wage quintile or the ‘higher managerial, administrative or professional’ occupation types tending to have highest life expectancy (Fig. 4). For women, there was 3.2 (95% CI: 0.4–5.6) years' difference in life expectancy between the wage quintiles with lowest and highest life expectancy and 3.3 (95% CI: 1.3–5.1) years' difference across occupational groups, whereas for men, these differences were 7.0 (95% CI: 3.5–9.8) years and 4.4 (95% CI: 3.2–5.5) years respectively (Fig. 4).

Across education groups, the pattern was slightly less clear. For both sexes, life expectancy is lowest for those with no qualifications as compared to all the other educational groups. Men in the ‘no qualifications’ group had lowest life expectancy by far of all socio-economic groups at 57.0 (95% CI: 56.2–57.9) years from age 20. For women, the longest life expectancy among educational groups is observed for those with apprenticeships: there is 5.4 (95% CI: 2.2–8.1) years longer life expectancy in this group compared to women with no qualifications (Fig. 4B). For men, on the other hand, life expectancy in the apprenticeship group is relatively short, and longest life expectancy is observed for those with degree-level education: 7.2 (95% CI: 3.0–10.1) years longer than those with no qualifications (Fig. 4A).

In addition, we compared our estimates to life expectancies from age 20 when calculated from aggregated area-level deprivation quintiles. These estimates were calculated from deprivation-specific life tables based on population-wide area-level Index of Multiple Deprivation (IMD) scores (Di Carlo et al., 2020). It wasn't possible to calculate aggregated statistics using the ONS-LS database due to data confidentiality restrictions. Using aggregated deprivation statistics, there was 7.2 years' difference in male life expectancy between highest and lowest quintiles, and 5.2 years' difference in female life expectancy. When compared to the differentials observed in the individual-level data across wage quintiles (7.0 years for men and 3.2 years for women), occupation groups (4.4 years for men and 3.3 years for women) and education groups (7.2 years for men and 5.4 years for women), the differences between men and women in terms of the overall extent of inequality generally appears to be similar when measured using individual-level data.

## Discussion

We have demonstrated that wide inequalities in adult mortality and life expectancy exist between both men and women of different individual-level socio-economic status. Our use of individual-level data allows us to clearly highlight important sex differences in the extent of inequality as well as sex differences in the SES dimensions (wage and education showing most extensive individual-level inequality for men, whereas wage inequalities for women were less pronounced). Consistently across both sexes, the group with “no qualifications” (as categorised by the education variable) experienced the lowest life expectancy, as compared to all other socio-economic groups. Individual-level data on both wage and education yielded differences in life expectation of a similar magnitude to the inequalities estimated from aggregated deprivation data. This suggests that differentials across individual socio-economic groups are as important as those previously measured between areas, but the underlying causes of such differentials are likely to differ and health policy may benefit from a differential approach, as well as further research to understand individual-level differences.

In terms of the similar patterns observed across individual wage and occupational groups, consistency between these socio-economic measures will be expected given the inherent link between income and job. Lower wage and certain occupational exposures are also likely to mean that an individual might have less time and resources to access healthcare, which could underlie the observed differences in mortality across these groups.

The inequalities observed across education groups followed a general pattern of higher mortality in the group with fewest qualifications, which is consistent with results of a study of adult mortality across educational groups in an Australian population (Korda et al., 2020), and with results of a large survey sample of English over-50s (Parker et al., 2020). However, a pattern across other education groups was less clear. Interestingly, while mortality was lowest for men with degrees and relatively high for men with apprenticeships, the opposite pattern was observed for women in these specific educational groups. Arguably, it is difficult to rank vocational training among educational groups, since careers associated with this type of training result in a wide range of incomes and quality of life (Office for National Statistics, 2016). For example, men with this type of education in England and Wales might commonly be associated with prolonged manual labour jobs such as construction or plumbing, which are associated with poorer health than the types of labour often associated with women in this same educational group, such as nurses or administrative roles (Office for National Statistics, 2016).

Unlike ecological measures of deprivation, which combine men and women's experience into a single geographical score, using individual data allows a direct comparison of inequalities between men and women. Wider inequalities across socio-economic groups were found for men than for women, and in the most extreme case, there was 7.0-years’ difference between the highest and lowest life expectancies for men across different wage quintiles compared to only 3.2-years’ difference for women across wage. It is likely, at least for a significant proportion of adults, that the influence of wage on overall health for women is tempered to some extent by a male partner's wage, which is likely to be higher, as seen in these data and elsewhere (Office for National Statistics, 2019a, Office for National Statistics, 2019b). This implies that some women on relatively low wages are actually experiencing a higher overall household income, and this is likely to at least partially explain the subtler differences seen across wage groups for women than for men. Our strategy to include a small proportion of individuals with missing socio-economic data by proxy using another adult in the household could dilute any such effect, however. Due to our focus on adult mortality, the majority of missing data was completed using data for a spouse or partner, and this method would potentially reduce differences observed between the sexes. However, we expect any such effect to be small, since only approximately 5% of cases were completed using another household adult. In general, we observed a higher proportion of missing data in older age groups in the cohort, which was compounded by older people being less likely to live with another adult from whom missing data could be completed. A sensitivity analysis excluding individuals with missing data reinforces the interpretation that the missing data strategy did not affect the results in terms of mortality differences observed between groups.

We also explored the impact of our missing data strategy in terms of treating the small proportion of ‘unemployed’ individuals in the dataset as missing occupation data, and therefore completing occupation using another household adult for these individuals, which affected approximately 3% of the total cohort. Previous research has reported higher mortality for unemployed or economically-inactive individuals, especially for men (Clemens et al., 2014), and the results of re-analysis of our data including ‘unemployed’ as a fourth occupational category are consistent with this. Life expectancy from age 20 for unemployed men was 50.6 years (95% CI: 49.4–52.1), and for women was 57.8 years (95% CI: 56.3–59.5), and the life expectancy estimates of the other occupational groups increased slightly (by 0.2–0.5 years) using this alternative analysis strategy.

Inequalities between individual-level socio-economic groups were of similar magnitude to those described with area-level deprivation data, in terms of the number of years’ difference in life expectancy across all groups (Bennett et al., 2018; Woods et al., 2005). In addition, both types of data give evidence of wider inequalities across men than women, however these sex differences are more pronounced when estimated using individual-level wage. This could be partially explained by aggregated deprivation statistics representing summarised data for an area, therefore combining data for all men and women. This strongly supports the hypothesis that individual-level factors underlying the association between socio-economic status and mortality are at least as important as area-level factors (Ingleby et al., 2020; Moscrop et al., 2020), and that ideally, each measure should complement the other in a simultaneous analysis.

Taken together, these results show that policies directed at reducing socio-economic inequalities in mortality would benefit from further research aimed at understanding the relative role of individual-and contextual circumstances in health outcomes. The use of individual-level data allows for a more detailed understanding of mortality differentials, in terms of increased ability to disentangle effects of different socio-economic variables and sex differences, which in turn allows for specific underlying causes of mortality differences to be targeted. Further research might be able to look at the combined effects of different socio-economic variables, for example, to determine if life expectancy of someone with no qualifications, but a relatively high income, is approximately in the middle of the estimates for each of these groups individually, or whether one aspect of socio-economic status is more influential than another. Similarly, further research could explore a combination of individual- and area-level measures, using a multi-level analysis approach to address more complex questions (Charvat et al., 2016; Diez-Roux, 1998; Rabe-Hesketh & Skrondal, 2012; Subramanian, 2004), for instance, how does the life expectancy of someone with no qualifications differ depending on if they live in an area with high levels of educational attainment compared to if they live in a relatively deprived area? This level of detail would enable increasingly targeted healthcare policy recommendations. Our analyses were carried out separately for each different socio-economic variable due to data access limitations with regards to accessing a dataset containing multiple different measures per individual, but such an analysis may be possible in future with a different dataset.

We acknowledge that individual SES and area-level deprivation are both time-varying exposures across the life course, and as such could change over the course of an individual's life as well as potentially having a reverse causal effect in the context here, where ill health could lead to changes in socio-economic group in some cases. This could also be an interesting direction of further research with respect to individual-level socio-economic circumstances. A comparison of mortality estimates reported here with the relative risk of death calculated between deprivation groups in UK data by Mackenbach et al. (Mackenbach et al., 2008), and the inequalities described in English over-50s by Parker et al. (Parker et al., 2020), suggests that individual-level socio-economic inequalities in mortality have persisted over recent years, although a detailed comparison is not possible due to the differences in data and analytical methods used. In addition, the differences observed here in terms of life expectancy across occupational groups are similar to those calculated using 2002–2006 data on a working-age sample (Johnson, 2011), suggesting little evidence for any reduction of these particular inequalities. Here, we have attempted to provide a snapshot of mortality and life expectancy focussed on socio-economic data from the 2011 census and mortality data from the same year, thus avoiding the problem of time-varying exposure. A subsequent census in England and Wales took place in March 2021, and so in time this data will be available to examine potential change in these inequalities in recent years.

In conclusion, our results illustrate wide inequalities in adult mortality and life expectancy between individual-level socio-economic groups, suggesting that inequalities in health outcomes are strongly linked to individual circumstances as well as healthcare resources at an area-level. These results clearly demonstrate that substantial inequalities in mortality cannot be fully understood using only area-level aggregate deprivation statistics. Further work focussing on the role of individual-level socio-economic circumstances in conjunction with area-based measures of deprivation in disease-specific areas of research will be useful in order to enable evidence-based policy recommendations to address these inequalities.

## Funding

This study was supported by a grant from the 10.13039/501100000269Economic and Social Research Council (ES/S001808/1) and partially by a programme grant from 10.13039/501100000289Cancer Research UK (C7923/A18525).

## Ethics approval

London School of Hygiene and Tropical Medicine Ethics Online Application 14600; approved February 01, 2018.

## Consent for publication

Permissions to publish were sought and agreed with the ONS in accordance with ONS practices.

## Data availability

Data are not publicly available but can be accessed via appropriate application to the ONS Longitudinal Study. This work contains statistical data from ONS which is Crown Copyright. The use of the ONS statistical data in this work does not imply the endorsement of the ONS in relation to the interpretation or analysis of the statistical data. This work uses research datasets which may not exactly reproduce National Statistics aggregates. All analysis code can be requested from the corresponding author.

## CRediT authorship contribution statement

**Fiona C. Ingleby:** Methodology, Formal analysis, Writing – original draft, Writing – review & editing, Visualization. **Laura M. Woods:** Conceptualization, Methodology, Writing – original draft, Writing – review & editing, Funding acquisition. **Iain M. Atherton:** Conceptualization, Methodology, Writing – original draft, Writing – review & editing, Funding acquisition. **Matthew Baker:** Writing – review & editing. **Lucy Elliss-Brookes:** Writing – review & editing. **Aurélien Belot:** Conceptualization, Methodology, Writing – original draft, Writing – review & editing, Funding acquisition.

## Declaration of competing interest

None to declare.

## Footnotes

Appendix A Supplementary data to this article can be found online at https://doi.org/10.1016/j.ssmph.2021.100815.
