# Supplementary material for: Describing socio-economic variation in life expectancy according to an individual's education, occupation and wage in England and Wales: An analysis of the ONS Longitudinal Study
Source: SSM Popul Health. 2021 May 8;14:100815. doi: 10.1016/j.ssmph.2021.100815 (PMC8131985; doi:10.1016/j.ssmph.2021.100815)
Supplement: Multimedia component 1 [file mmc1.docx]

**Supplementary materials**

**Table S1.** Distribution of LS members aged 20+ included in analysis over socio-economic groups, by sex. For occupation and education groups, the percentage of LS members per category is shown. For wage quintiles, individuals are divided equally into quintiles and the median weekly wage estimate (GDP) is shown with the standard deviation per quintile. Data source: ONS LS.

|  | **Men** | **Women** |
| --- | --- | --- |
| **Total** | **174,931** | **188,844** |
|  |  |  |
| **Occupation** |  |  |
| Managerial/Professional | 37% | 33% |
| Intermediate | 23% | 28% |
| Manual/Technical/Routine | 40% | 39% |
|  |  |  |
| **Education** |  |  |
| Degree-level or higher | 28% | 27% |
| A-levels | 13% | 12% |
| Apprenticeship/Vocational training | 13% | 7% |
| 5+ GCSEs | 12% | 16% |
| 1-4 GCSEs | 13% | 13% |
| No qualifications | 21% | 25% |
|  |  |  |
| **Wage** |  |  |
| Least deprived | £395 (40.5) | £276 (37.6) |
| Q2 | £299 (19.9) | £186 (21.1) |
| Q3 | £236 (17.5) | £137 (11.9) |
| Q4 | £184 (14.3) | £102 (9.5) |
| Most deprived | £120 (30.2) | £65 (14.1) |
|  |  |  |

**Figure S1.** Mortality rate for 1 person-year (log scale) for ages 20-100 for (A) men and (B) women. Red lines show the estimation (±95% confidence interval) based on a regression model with only age included; dashed black line shows the equivalent estimate from the publicly-available life tables for England and Wales, 2011. Shaded grey area shows ages 86-100, which were estimated by out-of-sample prediction using model coefficients (see text for details). Data source: ONS LS.
